# TUDCA Ameliorates Cognitive Impairment in APP/PS1 Mice by Modulating the Microbiota–Gut–Brain Axis

**DOI:** 10.3390/cimb48010087

**Published:** 2026-01-15

**Authors:** Minxia Zhan, Hui Chen, Xunzhong Fu, Shijin Tang, Xiaoxian Song, Henghua Li, Liancai Zhu, Bochu Wang

**Affiliations:** 1 Key Laboratory of Biorheological Science and Technology, Ministry of Education, College of Bioengineering, Chongqing University, Chongqing 400045, China; 20201901017@stu.cqu.edu.cn (M.Z.); 20161902090@cqu.edu.cn (S.T.); 2 Chongqing Academy of Chinese Materia Medica, Chongqing 400065, China; chenhui-520@126.com (H.C.); pugongying087728@163.com (X.S.); lhh8010_2002@163.com (H.L.); 3 School of Chemistry and Chemical Engineering, Chongqing University, Chongqing 400030, China; fuxunzhong@yaoyilk.com

**Keywords:** TUDCA, microbiota–gut–brain axis, Alzheimer’s disease, inflammation, TLR4/NF-κB/NLRP3

## Abstract

Tauroursodeoxycholic acid (TUDCA), a bile acid conjugate, has been suggested to improve cognition in models of Alzheimer’s disease (AD), although its underlying mechanisms remain unclear. This study aimed to evaluate the effects of TUDCA and its potential pathways in APP/PS1 mice. Behavioral tests, assessments of amyloid-β (Aβ) deposition, neuroinflammation, peripheral inflammatory responses, intestinal barrier integrity, and gut microbiota composition were performed, along with pseudo-sterile mouse experiments and fecal microbiota transplantation (FMT). The expression of genes related to the TLR4/NF-κB/NLRP3 pathway was also examined. TUDCA significantly ameliorated cognitive impairments, reduced Aβ accumulation, and suppressed inflammatory responses in both the central nervous system and peripheral tissues. It improved intestinal barrier function and reshaped gut microbial composition by reducing pro-inflammatory taxa. FMT demonstrated that TUDCA-modulated microbiota contributed to improved learning and memory in AD mice, whereas antibiotic-induced pseudo-sterility indicated that TUDCA also exerted cognitive benefits independent of gut flora. Moreover, TUDCA inhibited the activation of the TLR4/NF-κB/NLRP3 pathway. In conclusion, TUDCA alleviates AD-related cognitive deficits partly through modulation of the microbiota–gut–brain axis while also acting via microbiota-independent mechanisms, supporting its potential as a promising therapeutic strategy for AD.

## 1. Introduction

Alzheimer’s disease (AD) is a complex neurodegenerative disorder with multiple factors contributing to its development [1,2,3]. The rising rate of population aging has resulted in a global increase in the occurrence of AD, which has emerged as a significant issue in public health. At present, cholinesterase inhibitors (ChEIs) such as donepezil, levistan, galantamine, memantine, and other drugs that target a single enzyme have received approval for treating AD. Nevertheless, these drugs that target a specific pathway have limited and short-lived enhancements in cognitive function and can cause liver damage or cholinergic emergencies [4,5]. Hence, there are currently no therapies to prevent or slow down AD progression, highlighting the incomplete knowledge of disease mechanisms and the need for new drug targets.

AD is characterized by the presence of amyloid beta (Aβ) plaques, neurofibrillary tangles caused by the buildup of hyperphosphorylated tau protein within cells, and neuroinflammation [6]. Currently, these pathological characteristics serve as the primary explanations for the development of AD. Nevertheless, attempts to address AD by targeting the underlying causes of Aβ or tau have failed consistently in recent decades, indicating that the development of AD is likely to involve multiple factors and be significantly more intricate than solely the production of Aβ or tau. Despite numerous prior comprehensive investigations, the etiology of AD remains inadequately comprehended. Over the past few years, mounting proof suggests a correlation between changes in the gut microbiota and their metabolites and various neurological conditions, including Alzheimer’s disease. New scientific findings indicate that the gut microbiome has a two-way communication with the brain. Some researchers suggest that the human gut microbiome could be considered the ‘second brain’, playing a role in the development of AD and other neurodegenerative disorders [7,8,9,10]. Alterations in the intestinal environment can impact the production of various cytokines and chemokines by gut lymphocytes, including IL-1, IL-6, IL-17, IL-22, TNF-a, as well as chemokines and fractalkines. The presence of proinflammatory cytokines such as IL-1, IL-6, IL-17, and TNF-a may lead to brain damage [11,12,13]. Therefore, regulating gut microbes to improve cognition may be an effective way to interfere with the development of AD.

Recent pharmacological studies have demonstrated that TUDCA possesses antibacterial and anti-inflammatory properties, as well as the ability to enhance neuronal repair [14,15,16,17,18]. Moreover, it exhibits protective effects against various brain disorders [19,20,21]. This indicates that TUDCA may have a promising neuroprotective impact on AD. Bile acids have both direct antimicrobial effects on gut microbes and indirect effects through FXR-induced antimicrobial peptides [22,23,24]. This indicates that TUDCA may enhance cognitive impact on AD via participation of the microbiota–gut–brain axis.

Hence, this investigation was carried out in AD animal models to examine if TUDCA could enhance cognitive dysfunction via the participation of the microbiota–gut–brain axis. The findings indicated that TUDCA had a notable effect in alleviating cognitive impairments through the inhibition of Aβ plaque accumulation, enhancement of gut microbiota balance, restoration of the compromised intestinal barrier, and mitigation of the inflammatory reaction. TUDCA improves AD in various ways, and there are certain synergistic effects among these ways. TUDCA may inhibit the inflammation of AD through the TLR4/NF-*k*B/NLRP3 signaling pathway. The study provides new ideas and potential treatments for AD based on TUDCA’s potential therapeutic effects.

## 2. Materials and Methods

### 2.1. Chemicals and Reagents

Tauroursodeoxycholic acid, sodium salt-CAS 14605-22-2, Sigma Aldrich (Shanghai) Trading Co., Ltd. (Shanghai, China).

### 2.2. Animals and Treatments

APPswe/PSEN1dE9 transgenic mice (6-month-old, male) and C57BL/6J mice were provided by Cavins Laboratory Animal Co., Ltd. of Changzhou (Changzhou, China). The mice were housed in a controlled environment (20–24 °C, 55–65%, 12 h light-dark cycle) and had unrestricted access to food and water. All animal experiments were conducted according to the ethical rules of the Animal Experiment Chongqing Academy of Chinese Materia Medica (No. yls2021-7).

Following a week of habituation, the mice were randomly divided into three groups (8 mice per group): the AD group (Tg), the TUDCA group (Tg + TUDCA), and the C57BL/6J group (WT). For a duration of 6 months, the TUDCA group received a daily dosage of 30 mg/kg of TUDCA. The AD and WT groups were given the same dose of purified water.

A pseudo-aseptic AD mouse model was obtained by treatment with an antibiotic mixture (ABX). The specific methods are as follows: The antibiotic mixtures were selected as 1.25 mg/mL vancomycin, 2.5 mg/mL ampicillin, and 2.5 mg/mL metronidazole. Each AD model mouse was intragastric with 0.2 mL per day and continued intragastric for 7 days. Then, ABX treatment was given every 3 d during the experiment to ensure that the intestinal tract of AD mice was always in a state of limited bacteria during the feeding process.

In the fecal flora transplantation experiment (FMT), fecal samples of mice in the donor group (Tg + TUDCA group) were collected on a super-clean table, and then normal saline was added to prepare the fecal suspension. It was centrifuged at 4 °C at 800 rpm for 5 min to remove large particles of food residues. The fecal flora concentration was adjusted to 10^11^ CFU/L according to OD600. Mice in the recipient group (Tg + FMT group) were given 200 μL bacterial suspension each time by gavage, once every 4 days. The mice were divided into four groups (6 mice per group): Tg group (did not receive the 200 μL bacterial 110 suspension), Tg + TUDCA group (fecal samples of mice in the donor group), Tg + ABX + TUDCA group (mice were given 200 μL antibiotic mixture (ABX) + TUDCA), Tg + FMT group (mice were given 200 μL bacterial suspension from fecal samples of donor mice).

### 2.3. Behavioral Tests

Nesting Behavior Test: The mouse was placed in a clean cage specifically designed for observing nesting behavior. Two pieces of rectangular absorbent cotton, measuring 5 cm × 8 cm each, were evenly distributed within the cage. For a period of 24 h, the mouse was allowed to freely move around inside the cage. The nesting behavior of mice was evaluated and recorded, followed by a statistical analysis of nesting scores based on the following criteria (totaling 4 points): point 1 represents the lack of a noticeable nest; point 2 indicates the absence of an obvious nest, with nesting cotton occupying more than 1/3 of the cage area; point 3 signifies the presence of a prominent nest, where nesting cotton accounted for 1/4 to 1/3 of the cage area; and point 4 represents the visibility of a bowl-shaped nest or nesting cotton occupying less than 1/4 of the cage area.

The open field test (OFT) was conducted using an infrared detection system and a black inner open-air reaction box. The box was divided into 16 squares at the bottom, with 4 areas in the center and 12 areas on the edges. The actions of the mouse inside the container were carefully watched and documented for a period of 10 min in a quiet setting. Measured and recorded were the time spent in the central areas (s) and the distance traveled in the peripheral areas (mm).

The Morris water maze (MWM) test took place in a round tank filled with water and containing a hidden stationary platform located 1 cm beneath the water surface. During a 5-day training period, the mice were trained to locate the platform, and their escape latency was measured. Afterward, the platform was taken away, and the swimming path of mice, number of crossings, and the duration in the target quadrant were meticulously documented.

### 2.4. Thioflavin-S Staining

β-amyloid plaques were measured by Thioflavin-S staining (YuanyeBio-Technology Co., Ltd., Wuhan, China). Observations of the fluorescence expression of amyloid plaques were captured using a fluorescence microscope.

### 2.5. Immunofluorescence

The sections were immersed in 0.3% triton phosphate-buffered saline for 15 min and then subjected to an overnight incubation at 4 °C with primary antibodies against Occludin, Iba-1, and GFAP (Servicebio, Wuhan, China). Next, the sections were treated with the secondary antibody (Occludin: Alexa Fluor488 Goat Anti-Rabbit IgG, Abcam, Cambridge, UK; Iba-1, GFAP:HRP, Goat Anti-Rabbit IgG, Abcam, Cambridge, UK) and subsequently the nuclei were stained with DAPI for a duration of 5 min. Finally, the samples were sealed using an anti-fluorescence quencher. Ultimately, the fluorescence manifestation of the desired protein was witnessed and captured using the fluorescence microscope.

### 2.6. WGA and PAS Staining

The alterations in the intestinal mucous layer were identified using PAS staining (Servicebio, Wuhan, China) and WGA staining (Sigma-Aldrich, St. Louis, MO, USA) as per the guidelines provided by the manufacturer.

### 2.7. ELISA

The levels of Aβ1–40 and Aβ1–42 in the brain and IL–6, IL–1β, and LPS in serum were measured by ELISA (Elabscience Biotechnology Co., Ltd., Wuhan, China). The findings were reported as picograms per milliliter.

### 2.8. Luminex^®^ Assay

Luminex^®^ Assay (Bio-Rad Laboratories Co., Ltd, Shanghai, China)was used to measure the levels of pro-inflammatory and anti-inflammatory factors in both brain tissue and serum.

### 2.9. qRT-PCR

RNA was extracted from the brain and colon tissues of mice. TRIzol^®^ reagent (manufactured by Thermo Fisher, Waltham, MA, USA) was utilized. A spectrophotometer (DS-11, DeNovix, Wilmington, DE, USA) was used to measure the 260/280 ratio and determine the quantity and quality of RNA. Next, the Prime Script™ RT and SYBR^®^ fast qPCR master mixes were utilized in accordance with the guidelines provided by the manufacturer. The obtained cDNA was analyzed using the ABI-Step OnePlus Sequence Detection System (Applied Biosystems, Carlsbad, CA, USA) for qPCR analysis. The 2^-ΔΔCt^ method was utilized to calculate the levels of relative mRNA expression. The internal control chosen was β-actin. Table 1 displays the primers used for IL-1β, IL-6, TNF-α, NF-κB, TLR4, NLRP3, TNF-α, and β-actin mRNA.

### 2.10. Western Blot

The amount of protein was determined using the BCA protein detection kit. PAGE was used to perform constant pressure electrophoresis, using concentrated gel at 80 V for the gel separation and 120 V for the gel separation. Next, the proteins were transferred to the PVDF membrane and left to incubate overnight at 4 °C in various dilutions of TLR4 primary antibodies (1:1000, HuaBio, Hangzhou, China). Following the PBST wash, the proteins were exposed to HRP-labeled secondary antibodies (1:5000, Beyotime, Shanghai, China) for 1 h. Subsequently, visualized with ECL reagents, the chemiluminescence imaging system was used to observe. The quantitative analysis was conducted using Image J 1.52a software.

### 2.11. Microbiota Analysis

DNA from the colonic contents of mice was extracted using the OMEGA-soil DNA Kit (OMEGA Bio-Tek, Norcross, GA, USA). PCR was used to amplify the hypervariable region V3–V4 of the bacterial 16s rRNA gene. Quantification of the purified DNA gel products was performed using QuantiFluorTM-ST from Promega, Madison, WI, USA. The microbial genes were sequenced using Illumina NovaSeq, provided by Wekemo Tech Group Co., Ltd. in Shenzhen, China. The UPARSE tool 3.1.0 was utilized to group the high-throughput community sequences into operational taxonomic units (OTUs) with a similarity of 97% for the purpose of bioinformatics analysis of gut microbiota. Additionally, diversity and composition analysis were conducted for each group, and LEfSe analysis was performed with an LDA > 2.

### 2.12. Analysis of Statistics

Mean standard deviation was expressed by analyzing the statistical data with GraphPad Prism 8.0 software. Significance was determined using one-way ANOVA, with a threshold of *p* < 0.05.

## 3. Results

### 3.1. Effect of TUDCA on Cognitive Impairments in APP/PS1 Mice

**Figure 1:**
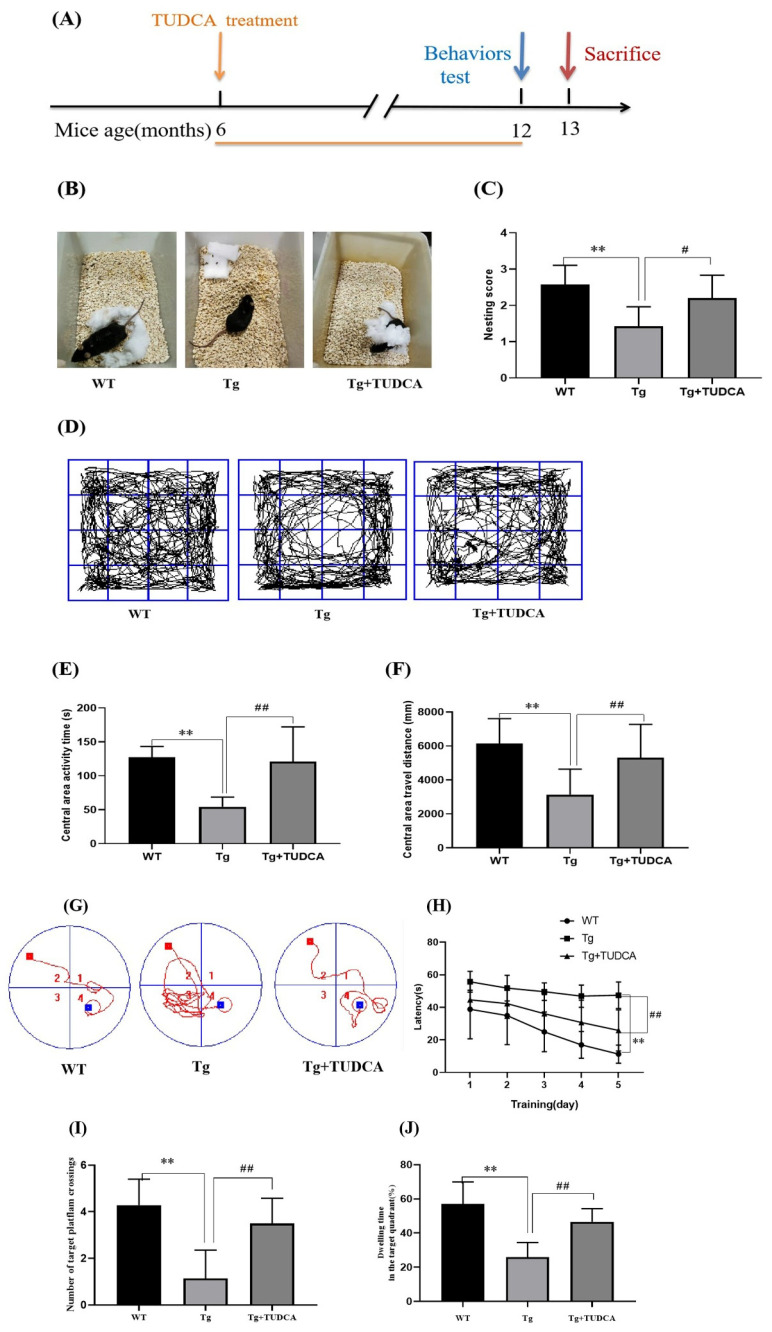
Effects of TUDCA on Tg mice’s cognitive deficits. (**A**) Treatment timeline for Tg mice with TUDCA, the orange line indicates that TUDCA treatment was administered during this period. (**B**) Photographs representing three groups of nesting. (**C**) Scores of nesting. (**D**) Representative pictures of mice in the open field. (**E**) Central area activity time. (**F**) Central area travel distance in OFT. (**G**) The representative locus diagram during the Morris water maze experiment, 1–4 represents the 1st - 4th quadrants, the red square indicates the starting point for the mouse’s releas, the blue square indicates the platform position, the red curve represents the movement route of the mouse. (**H**) The time it takes to escape in the Morris water maze test. (**I**) The count of intersections in the desired quadrant. (**J**) The percentage of time spent in the target quadrant was calculated by dividing the time spent in the target quadrant by the total time. Data are mean ± SEM. ** *p* < 0.01 vs. WT group, ^#^ *p* < 0.05, ^##^ *p* < 0.01 vs. Tg group.

To determine whether TUDCA exerts protective effects on cognitive decline in Alzheimer’s disease, we administered TUDCA to APP/PS1 mice beginning at 6 months of age, when early pathological features start to emerge. Behavioral assessments were conducted at 12 months of age to evaluate learning, memory, and exploratory activity, followed by tissue collection at 13 months for pathological and biochemical analyses (Figure 1A). This design allowed us to investigate both the long-term therapeutic impact of TUDCA and the progression of cognitive deficits in APP/PS1 mice. Figure 1B displays a representative image of mice nesting in each group during the nesting behavior test. Tg mice exhibited a significantly lower ability to construct complete nests compared to WT mice; however, mice treated with TUDCA constructed more complete nests and achieved higher nesting scores (*p* < 0.01, WT vs. Tg; *p* < 0.05, Tg vs. Tg + TUDCA, as shown in Figure 1C). The open field test revealed that Tg mice exhibited shorter durations of central area activity compared to WT mice, indicating increased anxiety. However, this anxious behavior was alleviated by TUDCA treatment. Significant differences were observed between WT and Tg (*p* < 0.01), as well as between Tg and Tg + TUDCA (*p* < 0.01). Furthermore, mice treated with TUDCA exhibited notably longer distances compared to Tg mice, while central distances of Tg mice were shorter than those of WT mice (*p* < 0.01, WT vs. Tg; *p* < 0.01, Tg vs. Tg + TUDCA, Figure 1D–F). Over the course of the five-day MWM experiment, every mouse progressively acquired the ability to search for concealed platforms, leading to a gradual reduction in their escape time. In Figure 1G, the locus plot indicates that the Tg mice exhibited erratic movement, while the mice treated with TUDCA moved within the target quadrant. After 2–5 days, Tg mice exhibited a longer escape latency than WT mice, whereas TUDCA-treated mice exhibited a notably shorter escape latency (Figure 1H). On the sixth day, there was a notable increase in platform crossings observed in the mice treated with TUDCA compared to the Tg group (Figure 1I). In comparison, mice treated with TUDCA spent a significantly longer amount of time in the target quadrant compared to Tg mice, as shown in Figure 1J. The results indicated that the TUDCA therapy ameliorated the cognitive impairments in the Tg mice.

### 3.2. Effect of TUDCA on Accumulation of Aβ in APP/PS1 Mice

**Figure 2:**
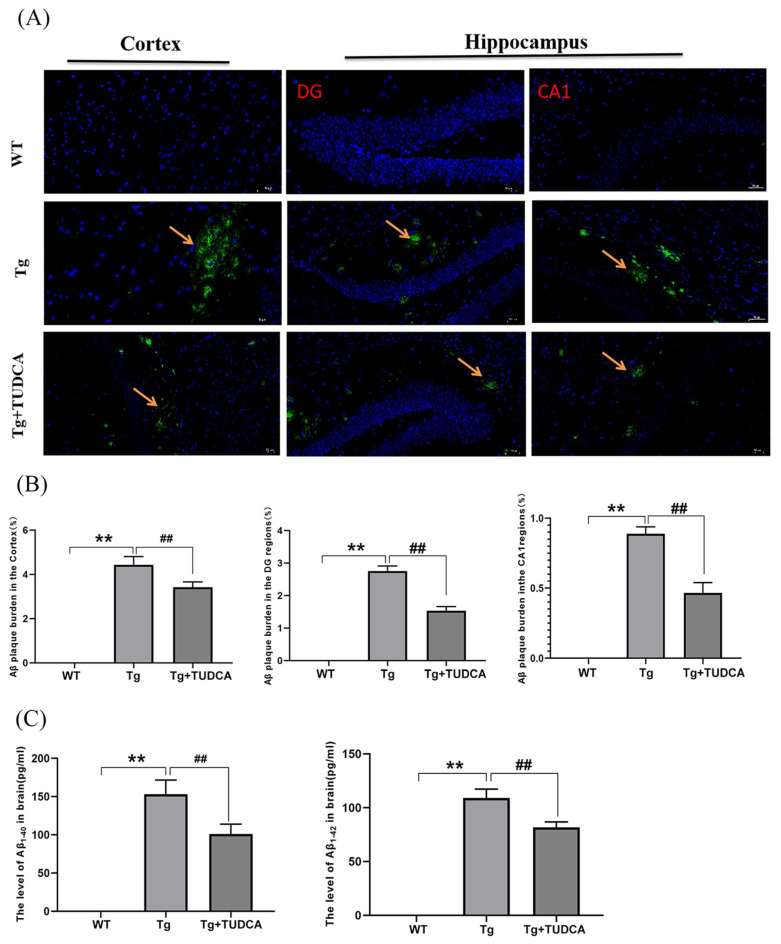
Treatment with TUDCA decreased the accumulation of Aβ plaques in Tg mice. (**A**) Representative images of Thioflavin S staining (green); scale bar = 50 µm. The yellow arrow indicates the plaque amyloid (green). (**B**) Thioflavin S staining quantitative analysis. (**C**) The concentration of Aβ1−40 and Aβ1−42 in the brain. Data are mean ± SEM. ** *p* < 0.01 vs. WT group, ^##^ *p* < 0.01 vs. Tg group.

Amyloid deposition is indicated by the presence of silvery yellow patches in Thioflavin-S staining. According to the findings, there was an increase in the silvery yellow patches in the Tg mice, whereas a decrease was observed in the TUDCA-treated mice (*p* < 0.01, Figure 2A,B). The concentrations of Aβ1-42 and Aβ1-40 in the brain tissues of Tg mice showed a significant increase, whereas the levels in the mice treated with TUDCA exhibited a significant decrease (*p* < 0.01, Figure 2C).

### 3.3. Effect of TUDCA on Neuroinflammation in APP/PS1 Mice

**Figure 3:**
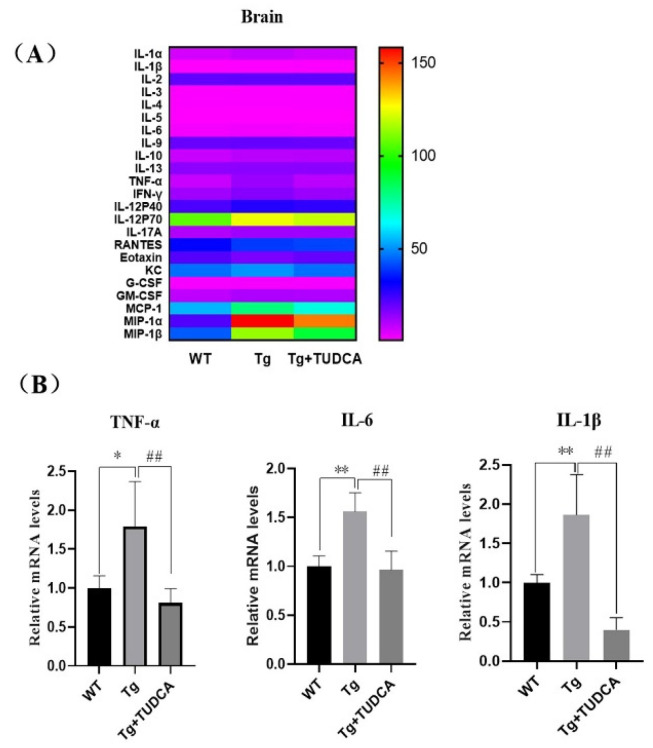
TUDCA suppressed inflammation in the brain. (**A**) The levels of inflammatory cytokines in the brain. (**B**) Relative levels of mRNA for TNF-α, IL-6, and IL-1β in brain tissue. Data are mean ± SEM. * *p* < 0.05, ** *p* < 0.01 vs. WT group, ^##^ *p* < 0.01 vs. Tg group.

In order to assess the effect of TUDCA on the inflammatory reaction in Tg mice, the Luminex^®^ Assay was utilized to analyze the levels of pro-inflammatory and anti-inflammatory agents in the brain tissue of the mice. The findings indicated that the concentrations of IL-1α, IL-β, IL-3, IL-6, IL-12P40, GM-CSF, TNF-α, TNF-γ, MCP-1, MIP-1α, and MIP-1β were elevated in Tg mice compared to WT mice. The levels of IL-1α, IL-6, TNF-α, and TNF-γ in the TUDCA-treated group were significantly lower compared to the Tg group (*p* < 0.01, Figure 3A). Additionally, we assessed the mRNA relative expression levels of TNF-α, IL-6, and IL-1β in brain tissue. In the Tg group, the mRNA levels of TNF-α, IL-6, and IL-1β showed a significant increase compared to those in WT mice (*p* < 0.01, Figure 3B). Moreover, the level of inflammation in mice treated with TUDCA was significantly reduced. The findings indicated that TUDCA could suppress inflammation in the brains of APP/PS1 mice.

### 3.4. Effect of TUDCA on Inflammation in the Peripheral Region of APP/PS1 Mice

**Figure 4:**
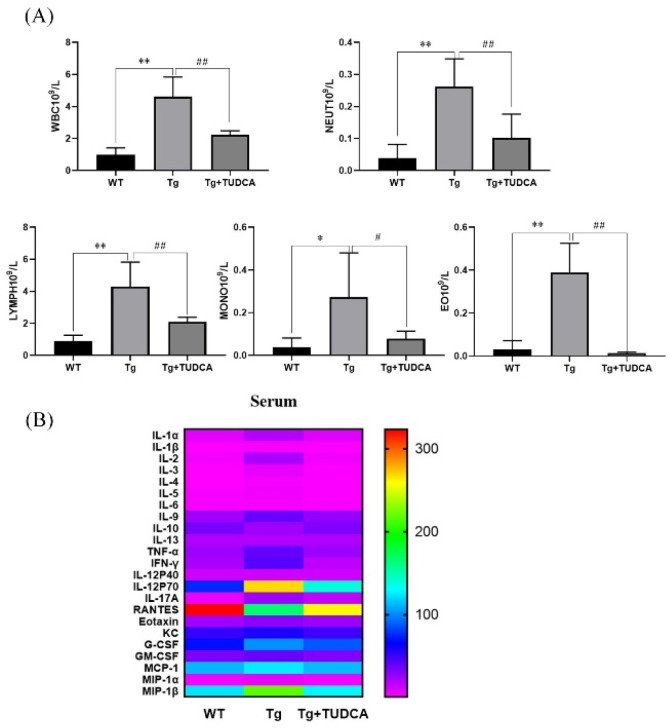
TUDCA inhibited peripheral inflammation in Tg mice. (**A**) TUDCA decreased the quantity of inflammatory cells in the serum of Tg mice. (**B**) TUDCA suppressed the secretion of inflammatory cytokines in the peripheral blood of Tg mice. Data are mean ± SEM. * *p* < 0.05, ** *p* < 0.01 vs. WT group, ^#^ *p* < 0.05, ^##^ *p* < 0.01 vs. Tg group.

To examine the effect of TUDCA on inflammation in the peripheral region of Tg mice, a routine blood analysis was used to evaluate the quantity of inflammatory cells in the peripheral blood of mice. A significant increase was observed in the count of peripheral blood leukocytes, neutrophils, and lymphocytes in the Tg group compared to the WT group (*p* < 0.01, Figure 4A). Additionally, there was an increase in the count of monocytes and eosinophilic cells (*p* < 0.05). Conversely, the count of leukocytes, neutrophils, lymphocytes, and eosinophils in the peripheral blood of mice in the TUDCA group showed a significant decrease compared to the Tg group (*p* < 0.01), with a decrease in monocytes as well (*p* < 0.05). Additionally, we assessed the levels of inflammatory factors in serum using Luminex technology for liquid suspension chip detection. In Tg mice, the levels of IL-1α, IL-1β, INF-γ, IL-17A, G-CSF, and MIP-1α were significantly increased (*p* < 0.05), and IL-2, IL-3, TNF-α, and MIP-1β were significantly increased (*p* < 0.01) compared to WT mice, while IL-10 and RANTES (CCL5) were significantly decreased (*p* < 0.01). Conversely, in TUDCA-treated mice, IL-1α, IL-1β, IL-2, IL-3, IL-5, IL-9, INF-γ, IL-17A, and MIP-1α were decreased (*p* < 0.05), while TNF-α and MIP-1β were significantly decreased (*p* < 0.01). However, IL-10 and RANTES (CCL5) were increased (*p* < 0.05) compared to Tg mice (Figure 4B). These findings indicate that TUDCA could suppress the infiltration of inflammatory cells and the release of pro-inflammatory factors while increasing the secretion of anti-inflammatory factors in the peripheral blood of Tg mice.

### 3.5. Effect of TUDCA on the Impairment of the Gut Barrier and Inflammation in APP/PS1 Mice

**Figure 5:**
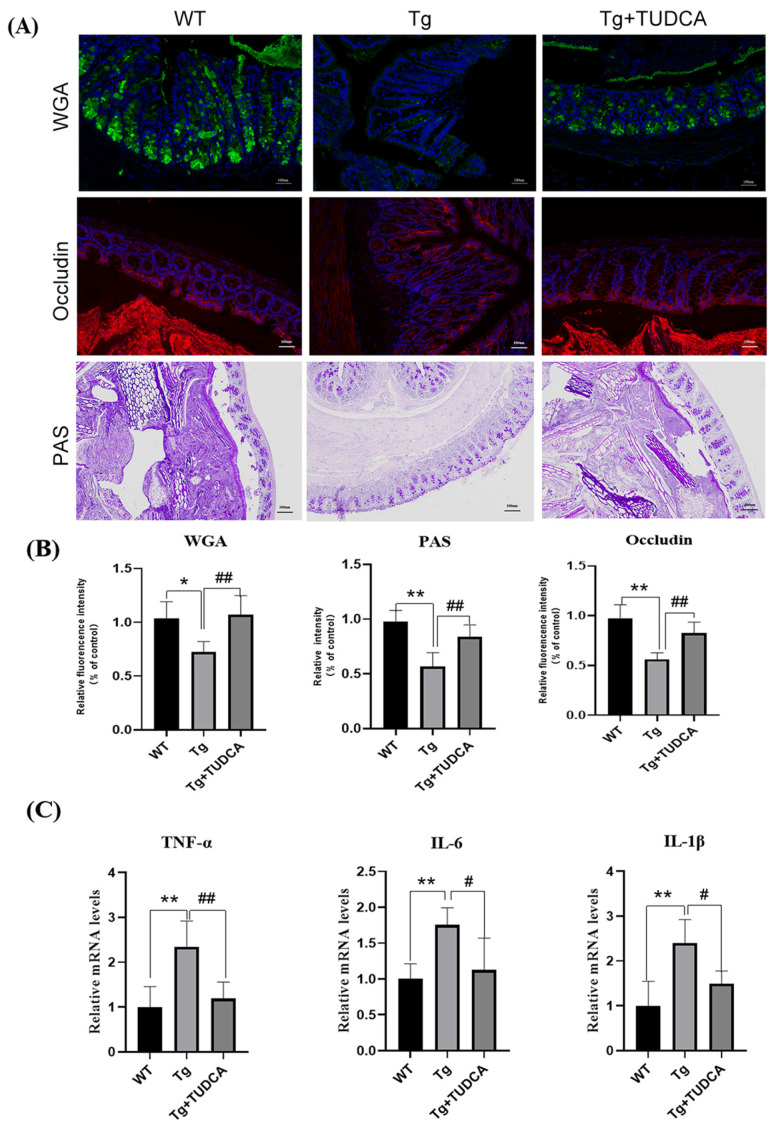
TUDCA enhanced the integrity of the intestinal barrier in mice. (**A**) Representative images of WGA (green), Occludin (red), and PAS (purple) staining; blue color represents DAPI, scale bar = 100 μm. (**B**) quantitative analysis of the staining. (**C**) qRT-PCR detected inflammatory cytokines in the colon tissue. Data are mean ± SEM. * *p* < 0.05, ** *p* < 0.01 vs. WT group, ^#^ *p* < 0.05, ^##^ *p* < 0.01 vs. Tg group.

To assess the impact of TUDCA on the colonic mucus layer in mice, we conducted FITC-WGA and PAS staining to examine the sugar chains of mucin glycoproteins. A decrease in mucin levels was observed in Tg mice compared to WT mice. However, there was a notable increase in mucin in the TUDCA group. To further assess the effect of TUDCA on the integrity of the colonic mechanical barrier, we utilized immunofluorescent staining to label the Occludin protein in colonic tissue. Tg mice exhibited a decrease in the fluorescence intensity of Occludin compared to WT mice. There was a significant increase in density in TUDCA-treated mice (*p* < 0.01, as shown in Figure 5A,B). To investigate the impact of TUDCA on intestinal inflammation in AD mice, we examined the alterations in pro-inflammatory factors at the transcriptional level in mouse colon tissue using qRT-PCR, which is commonly observed alongside damage to the intestinal barrier. Figure 5C displays the findings, indicating a notable rise in the mRNA levels of *IL-1β*, *IL-6*, and *TNF-α* in Tg mice when compared to WT mice. Moreover, there was a significant decrease in the mRNA levels of inflammatory markers in mice treated with TUDCA. The findings indicated that TUDCA plays a role in maintaining the integrity of the intestinal barrier in mice with AD. It is possible that TUDCA could effectively alleviate symptoms associated with AD by repairing the compromised intestinal barrier and mitigating the peripheral inflammatory response triggered by intestinal leakage.

### 3.6. Effect of TUDCA on the Microbiome in APP/PS1 Mice

**Figure 6:**
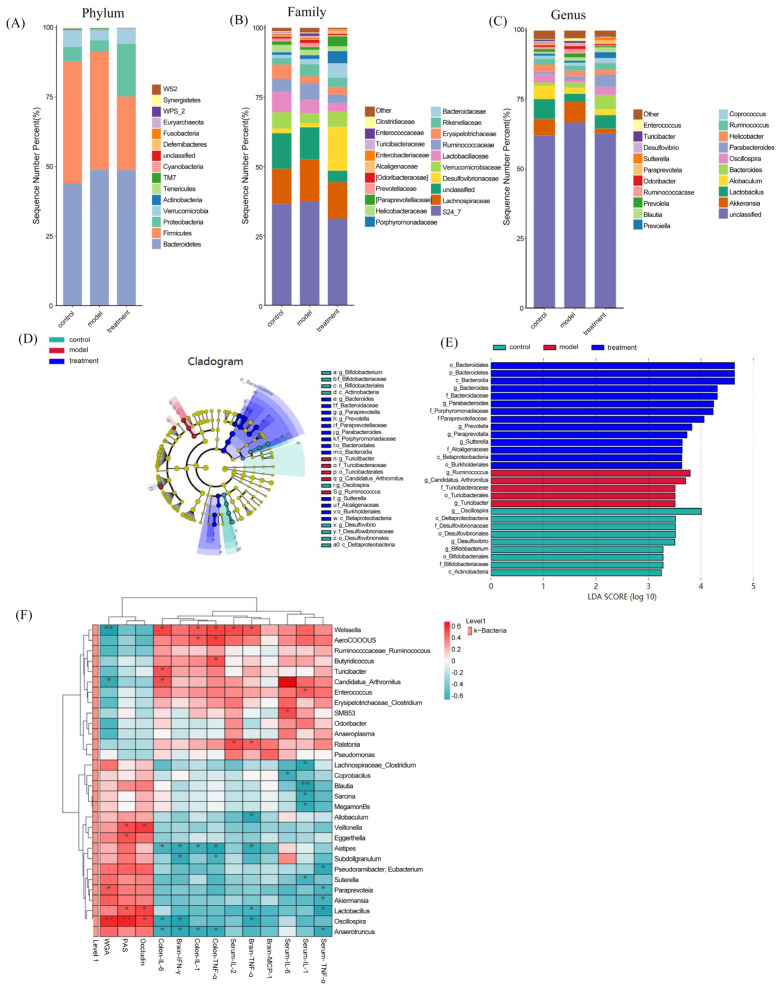
TUDCA modulates the intestinal microbiota composition in APP/PS1 mice. Relative abundance of gut microbiota at the phylum (**A**), family (**B**), and genus (**C**) levels. (**D**) LEfSe analysis showing taxa enriched in each group; the brightness of each dot reflects the magnitude of the effect size. Taxa enriched in the TUDCA-treated group exhibit positive LDA scores (treatment), while taxa enriched in WT mice (control)and APP/PS1 mice (model)show negative LDA scores. (**E**) Only taxa with an LDA score greater than two are displayed. (**F**) Heatmap illustrating the Spearman correlations between AD-related physiological parameters and altered bacterial genera among the experimental groups. Asterisks denote significant correlations after Spearman analysis combined with FDR correction (* *p* < 0.05, ** *p* < 0.01, *** *p* < 0.001).

Afterwards, we examined the impact of TUDCA on the composition of gut microbiota in APP/PS1 mice using 16s rRNA analysis. In Figure 6A, at the phylum level, the findings revealed that following TUDCA treatment in AD mice, there was a decrease in the abundance of *Firmicutes* and an increase in *Proteobacteria* compared to the AD group. At the family level, there was a reduction in the prevalence of *S24-7* in AD mice treated with TUDCA, whereas the prevalence of *Vibrio*, *Purpleomonas*, and *Bacteroideae* increased (Figure 6B). After TUDCA treatment, there was an increase in the prevalence of *Akkermansia*, *Lactobacillus*, *Oscillospira*, and *Paraprevotella* at the genus level in AD mice. The prevalence of *Prevoella* declined (Figure 6C). To compare the microbial variations among the three groups, the LDA effect size (LEfSe) was employed. This analysis identified a total of 28 distinct microorganisms, as depicted in Figure 6D,E. In the AD mice, the findings indicated that *Turicibacter* was among the prevailing bacterial clusters exhibiting notable variances, whereas the TUDCA group exhibited dominance of *Bacteroides* and *Paraphotella* bacteria. To elucidate the association between alterations in gut microbiota and physiological and biochemical markers associated with AD, we computed the Spearman correlation coefficient between them. According to the findings presented in Figure 6F, there was a notable association between alterations in gut microbiota at the genus level and the destruction of the intestinal barrier and inflammation. This suggests that an imbalance in gut microbiota plays a crucial part in the pathological progression of AD. At the genus level, the Spearman correlation analysis revealed a classification of intestinal microbiota into advantageous and detrimental bacteria. There are 20 beneficial bacterial groups (*Lactobacillus*, *Akkermansia*, *Paraprevotella*, *Oscillospira*, etc.). Intestinal integrity is positively associated with these factors, while inflammation is negatively associated with them. Nonetheless, there are detrimental bacterial clusters (*Enterococcus*, *Erysipelotrichaceae*, *Turicibacter*, *SMB53*, *Aerococcus*, *Weissella*, *Ruminococcus*, and *Ralstania*), which exhibit a positive association with inflammation while displaying a negative association with intestinal integrity. The findings indicate that the administration of TUDCA may modify the atypical microbial makeup in APP/PS1 mice’s intestines and effectively suppress the presence of proinflammatory microorganisms.

### 3.7. Effect of Intestinal Flora in the Treatment of TUDCA

To determine the role of intestinal flora in the treatment of TUDCA, we designed the following experiments: First, pseudo-aseptic AD model mice were constructed using an antibiotic mixture of 1.25 mg/mL vancomycin, 2.5 mg/mL ampicillin, and 2.5 mg/mL metronidazole. Each AD model mouse was given a gavage of 0.2 mL per day for 7 days. Then, TUDCA intervention was given, during which an antibiotic mixture was administered intragastrically every 3 d to ensure that the intestinal tract of AD mice was always in a state of a limited bacterial load during feeding. In addition, a fecal transplantation group was set up, and feces from the Tg + TUDCA group were transplanted into AD mice at 4 d intervals.

**Figure 7:**
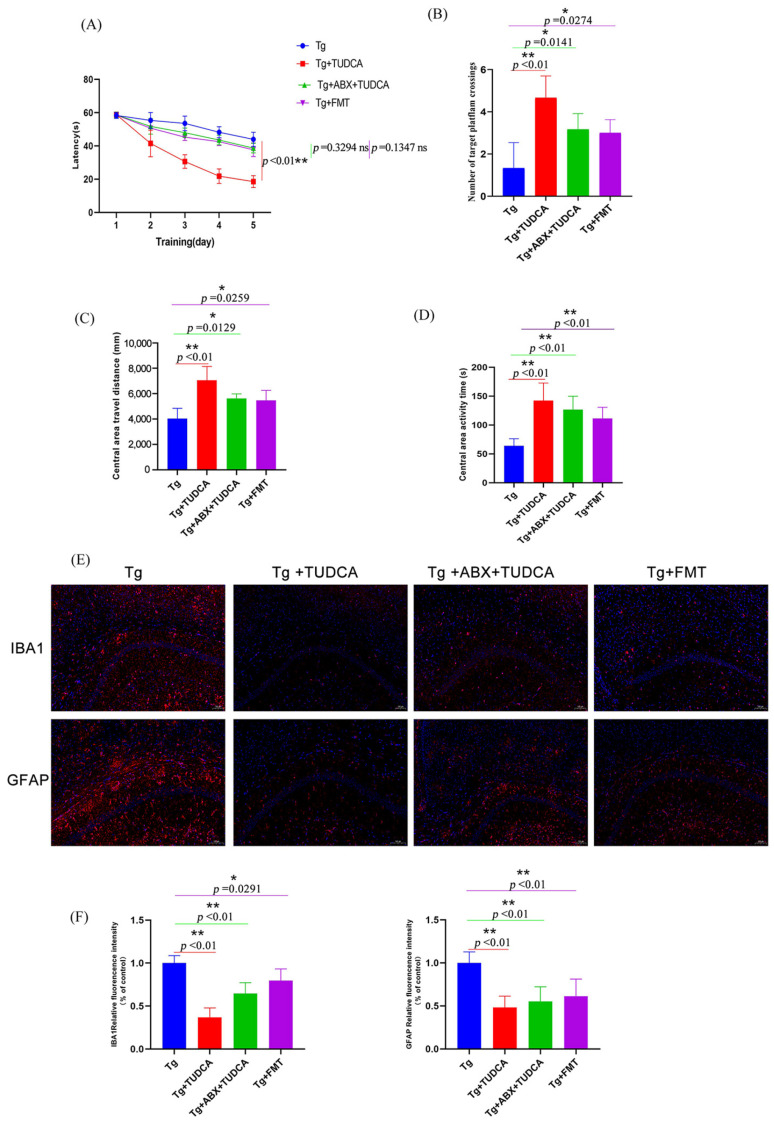
Behavioral assessments of mice in different treated groups and the microgliosis and astrocytosis status in the CAI region of the hippocampus. (**A**) The time it takes to escape in the Morris water maze test. (**B**) The count of intersections in the desired quadrant. (**C**) Central area travel distance in OFT. (**D**) Central area activity time. (**E**) Representative images of Iba-1 (red) and GFAP (red) staining, blue color represents DAPI; scale bar = 100 μm. (**F**) quantitative analysis of the staining. Data are mean ± SEM. * *p* < 0.05, ** *p* < 0.01 vs. Tg group, ns indicates no statistically significant difference.

After the intervention, behavioral differences were analyzed, and the results were shown in Figure 7A–D. Firstly, the Morris water maze was used to detect the learning and memory ability of mice in spatial orientation. The data showed that during the orientation navigation experiment in the first 5 days, compared with the Tg group, the escape incubation period of mice in the Tg + ABX + TUDCA group and the Tg + FMT group showed a trend of gradual shortening, but there was no statistical difference. These results indicated that the escape latency of AD mice was improved in both groups, but the effect was not obvious. On the 6th day, the platform was removed for a spatial exploration experiment. The results showed that compared with the Tg group, mice in the Tg + ABX + TUDCA group and the Tg + FMT group had significantly increased the frequency of crossing the virtual platform, but there was no significant difference between the two groups. Then, we carried out the open field tests, and the results show that both the Tg + ABX + TUDCA group and the Tg + FMT group exhibited longer durations and distances of central area activity compared to AD mice, indicating that this anxious behavior was alleviated, but the overall behavioral performance was not as good as the Tg + TUDCA group. FMT experiments showed that TUDCA could improve the spatial orientation disorder and improve the learning and memory ability of AD mice by regulating intestinal flora; the experiment of pseudo-sterile mice treated with antibiotics suggests that TUDCA may improve memory and cognition without intestinal flora.

Considering that one of the key features of AD is inflammation of the nervous system, the activation of microglia and astrocytes is an important reason for mediating injury and inflammation of the central nervous system and inflammation. The activation of glial cells is accompanied by an increase in number and a change in phenotype, a phenomenon known as reactive gliosis. Therefore, we observed the activation states of microglia and astrocytes. The results are shown in Figure 7E, F. Compared with the AD model group, the results showed less local proliferation and aggregation of glia in the brain after TUDCA treatment with pseudo-asepsis. In the FMT group, reactive gliosis was also improved, but the effect was still not as good as that of the AD + TUDCA group.

**Figure 8:**
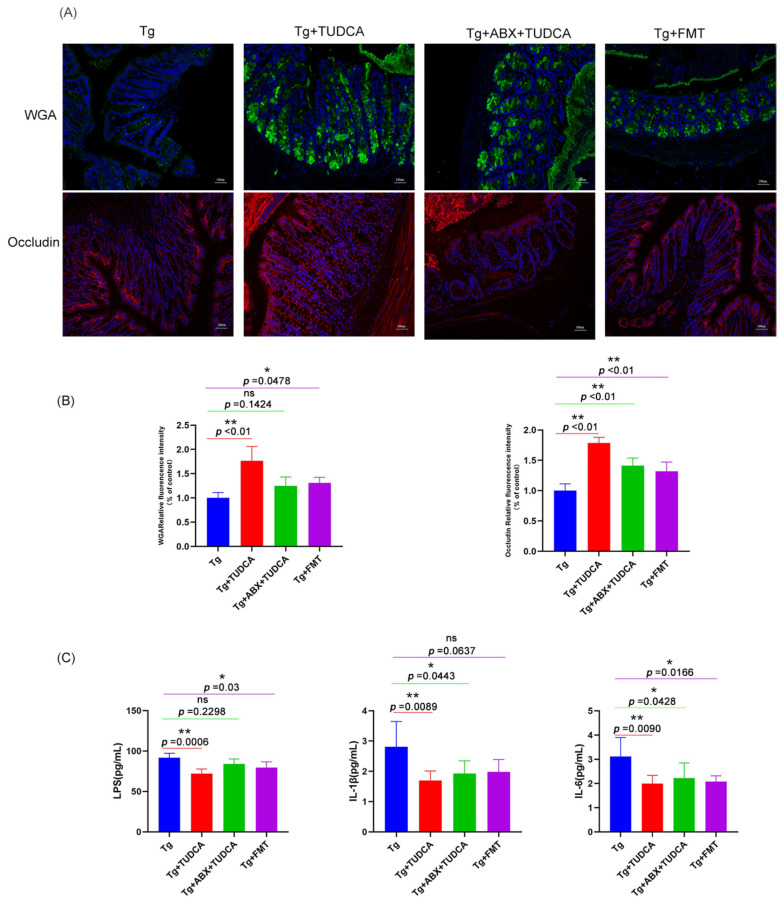
The integrity of the gut barrier in the colon of mice and pro-inflammatory cytokines release and LPS in the serum of mice in different treated groups. (**A**) Representative images of WGA (green) and Occludin (red) staining, blue color represents DAPI; scale bar = 100 μm. (**B**) quantitative analysis of the staining. (**C**) The expression levels of pro-inflammatory cytokines and LPS in serum were analyzed by ELISA. Data are mean ± SEM. * *p* < 0.05, ** *p* < 0.01 vs. Tg group, ns indicates no statistically significant difference.

We also tested the integrity of the intestinal barrier of mice in different treatment groups, and the results are shown in Figure 8A, B. Compared with the AD model group, after TUDCA treatment, the glycoproteins in the mucous layer of colon tissue of pseudo-sterile mice increased, indicating that the damage degree of the intestinal mucous layer was partially improved. In the fecal transplantation group, the glycoproteins in the mucous layer of the colon tissue of mice also showed an increasing trend. However, the overall effect is not as good as that of the AD + TUDCA group. The immunofluorescence staining results of intestinal tight junction protein Occludin also showed the same trend.

Then, we used ELISA to detect the serum inflammatory factors IL-1β, IL-6, and LPS. The results were shown in Figure 8C. Compared with the AD group, serum inflammatory factors and LPS of mice in both groups showed a certain degree of reduction with a statistical difference, but the overall effect was still worse than that of the AD + TUDCA group.

### 3.8. Effect of TUDCA on the TLR4/NF-κB/NLRP3 Pathway in APP/PS1 Mice

**Figure 9:**
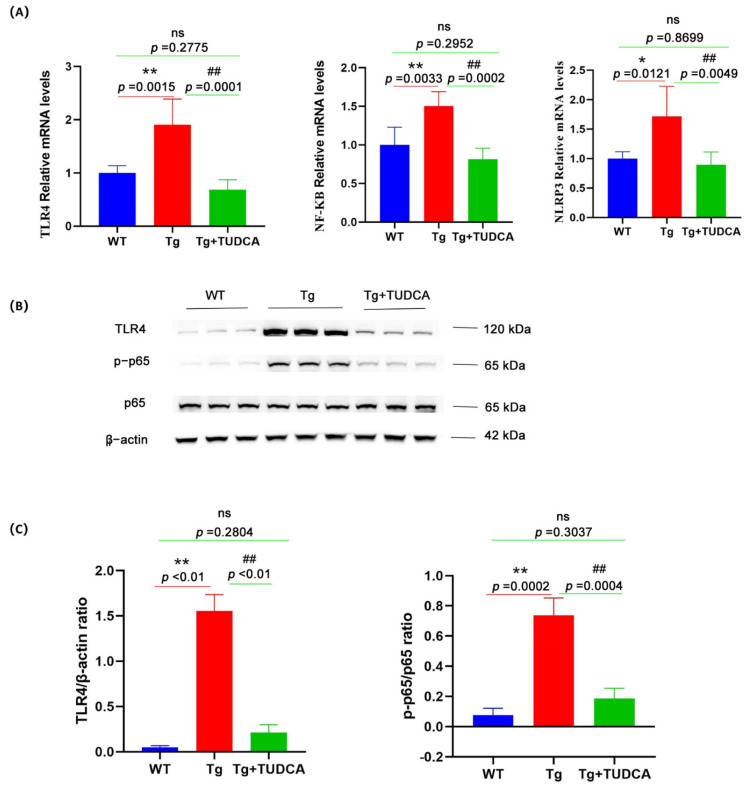
The effect of TUDCA on the TLR4/NF-κB/NLRP3 pathway in Tg mice. (**A**) The levels of mRNA expression for TLR4, NLRP3, and NF-κB were measured by qRT-PCR in brain tissue. (**B**) The protein expression level of TLR4, p65, and p-p65 was determined by Western blotting in brain tissue. (**C**) The panel includes semi-quantitative analyses for Western blot. Data are mean ± SEM. * *p* < 0.05, ** *p* < 0.01 vs. WT group, ^##^ *p* < 0.01 vs. Tg group, "ns" indicates no statistical difference.

The mRNA levels of TLR4, NF-κB, and NLRP3 were significantly raised in the brain tissue of mice compared to those in WT mice, whereas levels were reduced after TUDCA treatment in Tg mice (*p* < 0.05, Figure 9A). Moreover, the TLR4 protein level and the ratio of p-p65/p65 were notably increased in the brain tissue of Tg mice compared to WT mice, whereas they were significantly decreased in TUDCA-treated mice compared to Tg mice (*p* < 0.01, Figure 9B,C). These findings suggest that TUDCA may play a role in the regulation of the TLR4/NF-κB/NLRP3 pathway.

## 4. Discussion

Although the neuroprotective effects of TUDCA have been previously reported, its possible mechanisms against AD remain uncertain. In this study, the effects of TUDCA on anti-AD were evaluated, and its possible mechanisms were investigated in APP/PS1 mice. The findings of this research indicate that TUDCA has the potential to alleviate cognitive impairments through participation of the microbiota–gut–brain axis. This includes the inhibition of Aβ plaque accumulation, enhancement of dysbiosis, restoration of the impaired intestinal barrier, and suppression of the TLR4/NF-κB/NLRP3 pathway in order to mitigate the inflammatory reaction. TUDCA may improve AD in various ways, and there are certain synergistic effects among these ways.

Cognitive impairments are the primary features of AD. AD mice exhibited gradual cognitive impairment, including difficulties in memory and learning, which is a prominent feature of AD [25]. TUDCA in this research demonstrated the ability to reduce cognitive decline, wherein alterations in cognitive function are frequently accompanied by the pathological transformations associated with AD. The accumulation of amyloid in the brain was a significant factor contributing to the decline in cognitive function, which was notably increased in AD mice [26]. The findings from our study indicated that TUDCA had a significant impact on reducing amyloid accumulation in the APP/PS1 mice.

The intestinal barrier is a crucial defense mechanism in the human body and has a vital function in preserving intestinal balance. Intestinal barrier damage is often accompanied by a local intestinal inflammatory response [27]. Research has indicated that there is a strong correlation between various illnesses and the impairment of the intestinal barrier [28]. The impairment of the intestinal barrier will relocate the mutualistic bacteria and their toxins from the intestinal tract to the external tissues and organs, leading to the body being affected by internal microorganisms and their metabolic toxins, consequently triggering an overactive immune response [29]. Recent research indicates that AD patients have a significant amount of inflammatory signals originating from the gastrointestinal microbiota in their brains. This suggests that the intestinal barrier of AD patients might be compromised, leading to the release of these toxic molecules from the gut into the bloodstream and eventually reaching the brain [30,31,32]. Furthermore, research has indicated that inflammation is present alongside the impairment of colon tissue barrier function in mice with AD. The concentrations of inflammatory cytokines in both AD patients and model mice showed a significant rise, and the excessive presence of these cytokines worsened the pathological progression of AD [33]. In addition, impairments of intestinal barrier integrity may influence microbiota composition and mediate AD neuroinflammation [29,34]. Our results showed that the damage degree of the intestinal mucous layer and mechanical barrier was significantly improved in TUDCA-treated mice. Additionally, there was a notable decrease in the levels of inflammatory factors in both the brain and peripheral blood. These findings imply that TUDCA plays a crucial role in maintaining the integrity of the intestinal barrier in mice with AD. Furthermore, TUDCA has the potential to alleviate AD symptoms by repairing the compromised intestinal barrier and mitigating the peripheral inflammatory response resulting from intestinal leakage.

So how does TUDCA repair the damaged intestinal barrier and inhibit inflammation? Studies indicate that disruptions in the gut microbiota can result in heightened permeability of the intestinal barrier and an imbalance in metabolic substances. This can ultimately lead to the development of chronic inflammation in the peripheral region, potentially harming the blood–brain barrier, triggering neuroinflammation, damaging nerves, and ultimately resulting in neurodegeneration [35,36,37]. In the meantime, mounting evidence indicated the presence of abnormal microbiota in individuals with AD [38,39], which in turn could impact the makeup of microbiota and control the gut−brain connection [40,41,42,43]. Research has indicated that *Turicibacter* is associated with the levels of glial cell activation biomarkers and acts as an inflammatory bacterium, indicating the presence of brain inflammation. Additionally, *Bacteroides* and *Pallaputella* were found to have a positive correlation with the integrity of the intestinal barrier and the proper functioning of neurons and synapses. However, they exhibited a negative correlation with reactive gliosis and systemic inflammation [44,45,46]. In our study, we found that *Turicibacter* was among the major bacterial groups that exhibited notable variances in AD mice, whereas *Bacteroides* and *Paraphotella* were the predominant bacteria in the TUDCA group. The findings indicate that the administration of TUDCA may modify the abnormal microbiota in the intestines of APP/PS1 mice, effectively suppressing the presence of proinflammatory microbiota. TUDCA has the potential to control the imbalanced gut microbiota, suppress the chronic inflammatory response in the periphery caused by the rise in intestinal barrier permeability, subsequently reducing nerve damage caused by neuroinflammation, and ultimately leading to an improvement in the pathological symptoms of AD.

In order to further determine the role of intestinal flora in the treatment of TUDCA, we first constructed a pseudo-aseptic AD model in mice treated with antibiotics to explore whether TUDCA can improve related symptoms independently of intestinal flora. The results showed that in the pseudo-aseptic AD mouse model, to a certain extent, TUDCA treatment can also improve cognitive dysfunction, reduce reactive gliosis, repair damaged intestinal barrier, and reduce intestinal inflammation, but the therapeutic effect is not as good as that in the presence of intestinal flora. These results suggest that TUDCA may affect the pathological processes associated with AD through an independent pathway of intestinal flora. Subsequently, we transplanted the intestinal flora of the AD + TUDCA group into AD mice using the FMT method to explore whether the intestinal flora remodeled by TUDCA can improve the effect of AD. The results showed that FMT can also improve AD-related symptoms to a certain extent, but the effect is still not as good as the overall intervention effect. The above results show that TUDCA can improve AD in various ways, and there are certain synergistic effects among these ways. This effect is consistent with the overall concept of multi-link, multi-target, and synergistic action of many natural product drugs. And TUDCA can enter the brain through the blood–brain barrier and exert its neuroprotective effect in the previous study. In summary, the improvement effect of TUDCA on AD may be synergistic in various ways.

New evidence has emerged indicating that TLR4, a recognition receptor found in microglia, astrocytes, and neurons of the CNS, can rapidly detect various molecules associated with risk and activate the corresponding pathways for inflammation. This ultimately leads to neuroinflammation in AD, as the activation of TLR4 has been observed in AD patients. TLR4 is an important receptor in the inflammatory response process, activating the kernel factor NF-κB and inducing the activation of inflammatory mediators, thereby causing an inflammatory cascade reaction. Activation of the TLR4 signaling pathway can cause neuron denaturation and loss, thereby accelerating the occurrence and development of AD. NLRP3 is the most studied inflammatory body and is involved in the pathogenesis of AD. The NLRP3 inflammasome was overactivated in AD patients and APP/PS1 transgenic mice [47,48,49,50]. By suppressing the TLR4/NF-κB/NLRP3 pathway, it is possible to reduce neuroinflammation and neuronal death in the hippocampus of Aβ-induced AD rats [51]. Systemic LPS level is related to the permeability of the blood–brain barrier, acts on the blood–brain barrier, directly affects brain neurons or stimulates immunity, and participates in neuroinflammation or neurodegeneration. Systematic LPS can stimulate microglia and cause chronic neuroinflammation; TLR4 is the direct target of LPS activation, and LPS activates the TLR4/NF-kB signal in brain tissue [52,53]. In this study, TUDCA significantly reduced the levels of serum inflammatory factors IL-1β, IL-6, and LPS, and the levels of proteins and mRNA related to the TLR4/NF-κB/NLRP3 pathway. These results suggest that TUDCA may inhibit the TLR4/NF-κB/NLRP3 pathway against the inflammation of AD.

TUDCA has a part in the microbiota–gut–brain axis. Inflammation and neuroinflammation may be affected by TUDCA through interactions with systemic immune cells. Nevertheless, the precise mechanisms underlying the communication between microbiota, the gut, and the brain, as well as their potential impacts on behavior and cognition, remain largely unexplored. Graphical abstract showcased our theory on how TUDCA enhances cognitive function via participation of the microbiota–gut–brain axis.

## 5. Conclusions

To summarize, our research revealed that TUDCA has a part in the microbiota–gut–brain axis to alleviate cognitive deficits. TUDCA improves AD in various ways, and there are certain synergistic effects among these ways. These mechanisms include inhibiting the buildup of Aβ plaques, enhancing gut microbial balance, restoring the impaired intestinal barrier, and possibly suppressing the TLR4/NF-κB/NLRP3 pathway to decrease inflammation of AD. Taken together, TUDCA could potentially serve as a viable therapeutic approach for preventing and treating AD.

## Figures and Tables

**Table 1:** Nucleotide sequence of primers used in qRT-PCR.

Gene	Forward Primers	Reverse Primers
*TNF-α*	CGCTGAGGTCAATCTGC	GGCTGGGTAGAGAATGGA
*IL-6*	ACAGAAGGAGTGGCTAAGGA	AGGCATAACGCACTAGGTTT
*IL-1β*	AGTTGACGGACCCCAAA	TCTTGTTGATGTGCTGCTG
*NF-κB*	CTGCACTCTATGGCTCAGG	GGGACAGCGACACCTTT
*TLR4*	CTTTGCTTCCTTGGTGTTG	ATGATTCTCCTCTTCTTCACG
*NLRP3*	GACTGGCAAAAGGCTGTG	AGTTTCTCCAAGGCTACCG
*β-actin*	CCTCACTGTCCACCTTCC	GGGTGTAAAACGCAGCTC

## Data Availability

The original contributions presented in this study are included in the article. Further inquiries can be directed to the corresponding authors.
